# *ADRB2* Polymorphisms (rs1042713 and rs1042714) and Blood Pressure Response to the Cold Pressor Test in Combat Athletes and Non-Athletes

**DOI:** 10.3390/ijms26041765

**Published:** 2025-02-19

**Authors:** Marek Sawczuk, Agata Gąsiorowska, Agnieszka Maciejewska-Skrendo, Monika Chudecka, Katarzyna Kotarska, Patrizia Proia, Jolanta Marszałek, Paulina Małkowska, Katarzyna Leźnicka

**Affiliations:** 1 Institute of Physical Culture Sciences, University of Szczecin, 70-453 Szczecin, Poland; agnieszka.maciejewska-skrendo@usz.edu.pl (A.M.-S.); monika.chudecka@usz.edu.pl (M.C.); katarzyna.kotarska@usz.edu.pl (K.K.); paulina.malkowska@usz.edu.pl (P.M.); katarzyna.leznicka@usz.edu.pl (K.L.); 2 Faculty of Physical Culture, Gdansk University of Physical Education and Sport, 80-336 Gdansk, Poland; 3 Faculty of Psychology in Wroclaw, SWPS University, Aleksandra Ostrowskiego 30b, 54-238 Wroclaw, Poland; agasiorowska@swps.edu.pl; 4 Sport and Exercise Sciences Research Unit, Department of Psychology, Educational Science and Human Movement, University of Palermo, 90144 Palermo, Italy; patrizia.proia@unipa.it; 5 Faculty of Rehabilitation, Józef Piłsudski University of Physical Education in Warsaw, Marymoncka 34, 00-968 Warsaw, Poland; jolanta.marszalek@awf.edu.pl; 6 Doctoral School, University of Szczecin, 70-384 Szczecin, Poland

**Keywords:** combat athletes, young adults, cold pressor test, blood pressure, *ADRB2*, Gly16Arg, Glu27Gln, rs1042713, rs1042714

## Abstract

Adrenergic receptors (AR) play a vital role in cardiovascular system regulation. The *ADRB2* gene, encoding the β2-AR receptor, has genetic variability potentially impacting blood pressure (BP) regulation. Evidence for such associations has been inconsistent. This study investigates the relationship between two *ADRB2* polymorphisms (rs1042713, Gly16Arg, and rs1042714, Glu27Gln) and BP changes during the cold pressor test (CPT) in young, healthy men, including combat athletes. The study included two groups: combat athletes and non-athlete students. BP (systolic, SBP; diastolic, DBP) was measured at rest and at pain tolerance during CPT. Genetic analysis was conducted for rs1042713 and rs1042714 polymorphisms. Athletes had higher SBP and DBP than students, with both values increasing during pain tolerance compared to rest. Differences in BP responses during CPT were genotype-dependent. Students with the Gly16Gly16 genotype had significantly higher SBP than Arg16 allele carriers, while this variation was not observed in athletes. Athletes with the Glu27 allele exhibited higher SBP than 27Gln homozygotes, unlike students. Gly16 and Glu27 alleles are linked to elevated stress-induced BP responses in young Polish men. However, BP regulation involves multiple genetic and environmental factors not explored in this study.

## 1. Introduction

Adrenergic receptors (AR), particularly β-adrenergic receptors (β-AR), play a crucial role in the regulation of the cardiovascular system. The β_1_ and β_2_ subtypes are especially important in controlling heart rate (HR) and myocardial contractility, directly affecting cardiac performance [1]. In addition to mediating vasodilatory responses to adrenergic agonists, β_2_-AR also modulates renal function by regulating salt and water excretion, which is essential for maintaining proper blood pressure (BP). Research on β-AR and their genes is vital for understanding the molecular mechanisms underlying BP regulation and overall cardiovascular function [2].

Genetic variability in the gene *ADRB2* encoding the β_2_-AR receptor significantly impacts its functional role in the cardiovascular system. Polymorphisms rs1042713 (G>A, Gly16Arg) and rs1042714 (G>C, Glu27Gln) are among the most studied variants of the *ADRB2* gene [3]. The rs1042713 polymorphism results in a substitution of glycine with arginine at position 16, which can affect receptor desensitization and downregulation. This variation has been linked to altered responses to β-agonists and may influence susceptibility to hypertension and asthma. The rs1042714 polymorphism, involving a substitution of glutamic acid with glutamine at position 27, has been associated with enhanced receptor downregulation and variations in vasodilation efficiency [4]. It is widely suggested that these polymorphisms can modulate cardiovascular and metabolic responses, thereby contributing to individual differences in BP regulation and risk of cardiovascular diseases [1,5].

The cold pressor test (CPT) is a widely used and thoroughly validated method that induces systemic stress. It involves the immersion of the hand into an ice water container for a period of time, usually one minute [6]. The CPT has been employed across various disciplines to examine the effects of stress on pain, including pain threshold and pain tolerance, as well as on cardiovascular reactivity, specifically changes in BP and HR [7,8].

To our best knowledge, no studies have analyzed the impact of the CPT on cardiovascular parameters such as systolic (SBP) and diastolic BP (DBP) in relation to the genetic variability within the *ADRB2* gene in young healthy men, including combat athletes.

We think that understanding the interactions between rs1042713 and rs1042714 polymorphisms and cardiovascular responses to stress offers substantial benefits, particularly in the context of personalized cardiovascular risk assessment for individuals exposed to acute stress. This research can expand the understanding of cardiovascular adaptations in athletes, highlighting how genetic predispositions may contribute to distinct hemodynamic profiles compared to non-athletes. Moreover, investigating genetic variants associated with blood pressure regulation, including the rs1042713 polymorphism, could provide important insight into receptor regulation mechanisms under stress, thereby elucidating interindividual differences in hemodynamic responses. Additionally, the early detection of genetic susceptibility to hypertension through *ADRB2* polymorphisms facilitates timely and targeted interventions, while personalized training and healthcare strategies tailored to an individual’s genetic profile can optimize cardiovascular performance and mitigate potential health risks. Finally, these findings may contribute to the field of pharmacogenomics by elucidating how genetic variations in adrenergic signaling may influence the efficacy of cardiovascular treatments, such as β-blockers, thereby promoting more precise and effective pharmacological interventions.

Therefore, the aim of our study was to find a potential association of two selected *ADRB2* gene polymorphisms (rs1042713 and rs1042714) with SBP and DBP measured during the CPT in two groups of young healthy men (a group of combat athletes and non-athletes—a group of unrelated university students).

## 2. Results

The results of the anthropometric measurements together with the results of the CPT are shown in Table 1. The athletes were older and smaller compared to the students. There was no significant difference in body mass. The body mass index (BMI) indicated a normal weight for all study participants, and the differences between the groups were mainly due to differences in height. In addition, the athletes had a higher pain tolerance than the students, but the two groups did not differ in pain threshold.

In Table 2, we present the results of a multilevel regression examining SBP as a function of study group, pain threshold, and pain tolerance using the CPT and the different genotypes of *ADRB2* polymorphisms rs1042713 and rs1042714. The model explained R^2^ = 8.5% of the variance in SBP. We found a significant effect of a group such that athletes, on average, had higher SBP than students. We also found an effect of time of measurement, such that SBP was significantly higher when measured at the time of pain tolerance than when measured before CPT. We found no significant interaction between the study group and the measurement time, suggesting that, on average, the two groups performed the same during CPT. However, we found significant interactions between the group and the rs1042713 polymorphism and between the group and the rs1042714 polymorphism, suggesting that participants in the two groups had different levels of SBP during CPT depending on genotype. We also found significant interactions between the timing of measurement and the rs1042714 polymorphism, suggesting that participants with different genotypes responded differently to the CPT, regardless of whether they were students or athletes. None of the three-way interactions were significant.

**Figure 1:**
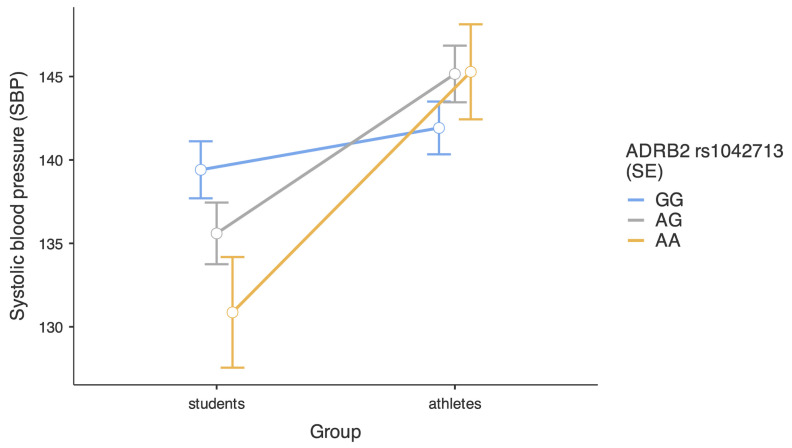
Level of systolic blood pressure in students and athletes depending on *ADRB2* rs1042713 polymorphisms. Bars represent standard errors.

Further, we decomposed the significant two-way interactions we found in the main multilevel regression. Figure 1 presents a decomposition of the interaction between the study group and the rs1042713 polymorphism. Analysis of simple effects revealed that the athletes demonstrated similar SBP, independent of their genotype, respectively, b = 3.24, SE = 2.40, 95%CI [−1.48, 7.95], t = 1.35, *p* = 0.178 for AG-GG comparison and b = 3.36, SE = 3.54, 95% CI [−3.59, 10.32], t = 0.95, *p* = 0.342 for the AA-GG comparison. In the group of students, we found a significant difference between those with AA and GG genotypes, b = −8.55, SE = 4.11, 95%CI [−16.62, −0.47], t = −2.08, *p* = 0.038, but not between those with AG and GG genotypes, b = −3.81, SE = 2.56, 95% CI [−8.84, 1.21], t = −1.49, *p* = 0.137. An alternative decomposition of the same interaction revealed that athletes and students did not differ in their SBP if they had the GG genotype, b = 2.51, SE = 2.33, 95% CI [−2.07, 7.09], t = 1.08, *p* = 0.282. However, athletes demonstrated higher SBP than students if they had an AG genotype, b = 9.56, SE = 2.51, 95%CI [4.62, 14.49], t = 3.81, *p* < 0.001, and if they had an AA genotype, b = 14.42, SE = 4.37, 95%CI [5.82, 23.01], t = 3.30, *p* = 0.001.

**Figure 2:**
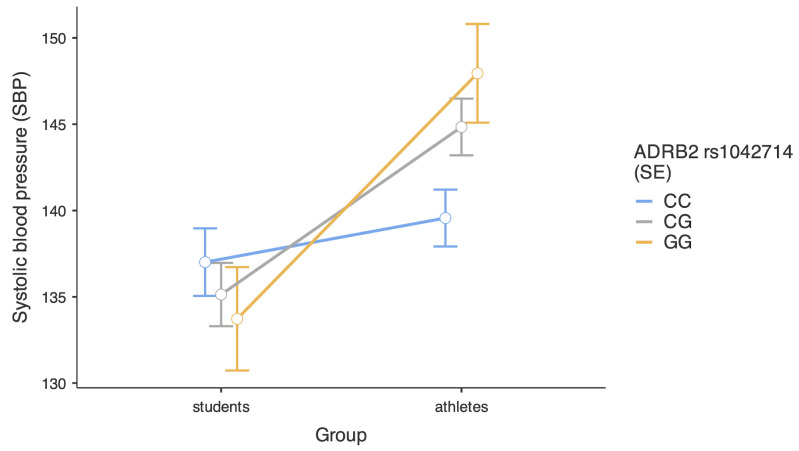
Level of systolic blood pressure in students and athletes depending on *ADRB2* rs1042714 polymorphisms. Bars represent standard errors.

Figure 2 presents a decomposition of the interaction between the study group and the rs1042714 polymorphism. Analysis of simple effects revealed that the students demonstrated similar SBP, independent of their genotype, respectively, b = −1.88, SE = 2.90, 95%CI [−7.59, 3.83], t = −0.65, *p* = 0.518 for the CG-CC comparison and b = −3.29, SE = 3.92, 95%CI [−11.00, 4.42], t = −0.84, *p* = 0.402 for the GG-CC comparison. In the group of athletes, we found a significant difference between those with CG and CC genotypes, b = 5.27, SE = 2.45, 95%CI [0.46, 10.09], t = 2.15, *p* = 0.032, and between those with GG and CC genotypes, b = 8.38, SE = 3.58, 95%CI [1.35, 15.42], t = 2.34, *p* = 0.020. An alternative decomposition of the same interaction revealed that athletes and students did not differ in their SBP if they had the CC genotype, b = 2.55, SE = 2.56, 95%CI [−2.46, 7.58], t = 1.00, *p* = 0.319. However, athletes demonstrated higher SBP than students if they had a CG genotype, b = 9.71, SE = 2.46, 95%CI [4.87, 14.54], t = 3.94, *p* < 0.001, and if they had a GG genotype, b = 14.22, SE = 4.14, 95%CI [6.08, 22.37], t = 3.43, *p* < 0.001.

**Figure 3:**
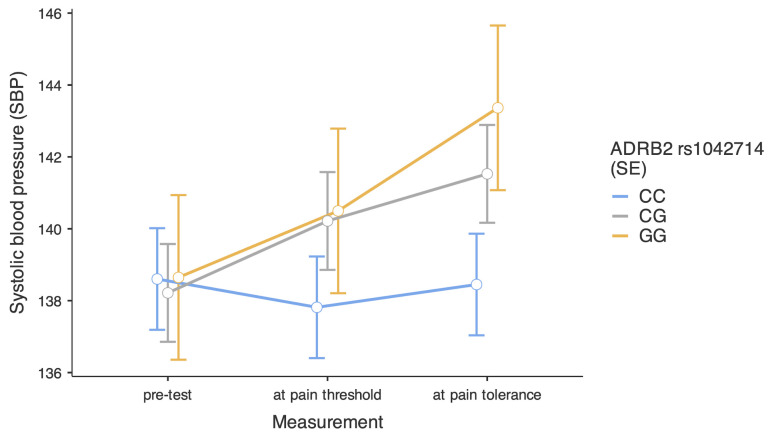
Level of systolic blood pressure depending on the moment in the cold pressor test and *ADRB2* rs1042714 polymorphisms. Bars represent standard errors.

Finally, we decomposed an interaction between the moment of SBP measurement and the rs1042714 polymorphism (see Figure 3). Analysis of simple effects revealed that people with the CC genotype had similar SBP independently of the moment in the CPT, respectively, b = −0.79, SE = 1.05, 95%CI [−2.84, 1.26], t = −0.75, *p* = 0.451 for the pre-test vs. pain threshold comparison, and b = −0.15, SE = 1.05, 95%CI [−2.21, 1.90], t = −0.15, *p* = 0.883 for the pre-test vs. pain tolerance comparison. In the group of participants with CG genotype, we found significant increases in SBP, respectively, b = 2.00, SE = 1.01, 95%CI [0.03, 0.98], t = 1.99, *p* = 0.047 for the pre-test vs. pain threshold comparison, and b = 3.31, SE = 1.01, 95%CI [1.34, 5.29], t = 3.29, *p* = 0.001 for the pre-test vs. pain tolerance comparison. Finally, in the group of participants with GG genotype, we did not find a significant increase in SBP at pain threshold, b = 1.85, SE = 1.69, 95%CI [−1.47, 5.17], t = 1.09, *p* = 0.275, while the increase at pain tolerance was significant, b = 4.72, SE = 1.69, 95%CI [1.39, 8.04], t = 2.79, *p* = 0.005.

In Table 3, we present the results of a multilevel regression examining DBP as a function of study group, pain threshold, and pain tolerance using the CPT and the different genotypes of *ADRB2* polymorphisms rs1042713 and rs1042714. The model explained R2 = 3.3% of the variance in DBP. We found a significant effect of a group such that athletes, on average, had higher DBP than students. We also found an effect of time of measurement, such that DBP was significantly higher when measured at the time of pain tolerance and pain threshold than when measured before CPT. The only significant interactions we found were those between the timing of measurement and the rs1042714 polymorphism, suggesting that participants with different genotypes responded differently to the CPT, regardless of whether they were students or athletes.

**Figure 4:**
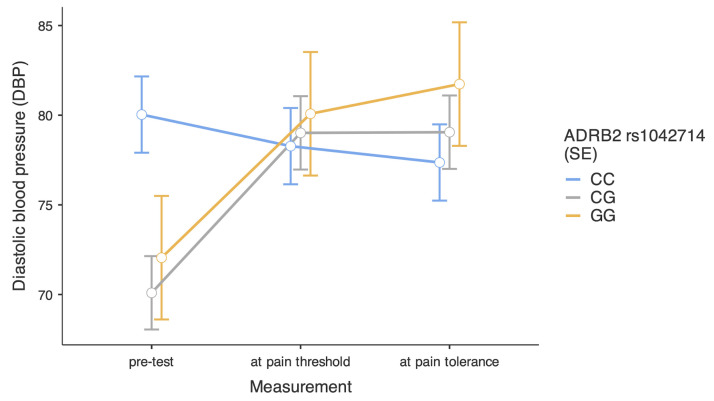
Level of diastolic blood pressure depending on the moment in the cold pressor test and *ADRB2* rs1042714 polymorphisms. Bars represent standard errors.

We further decomposed this interaction by investigating the simple effects (see Figure 4). As for the SBP, we found that people with the CC genotype had similar DBP independently of the moment in the CPT, respectively, b = −1.76, SE = 2.73, 95%CI [−7.11, 3.59], t = −65, *p* = 0.519 for the pre-test vs. pain threshold comparison, and b = -2.67, SE = 2.73, 95%CI [−8.02, 2.68], t = −0.98, *p* = 0.327 for the pre-test vs. pain tolerance comparison. In the group of participants with the CG genotype, we found significant increases in DBP, respectively, b = 8.92, SE = 2.62, 95%CI [3.77, 14.07], t = 3.40, *p* < 0.001 for the pre-test vs. pain threshold comparison, and b = 8.96, SE = 2.62, 95%CI [3.81, 14.11], t = 3.42, *p* < 0.001 for the pre-test vs. pain tolerance comparison. Finally, in the group of participants with the GG genotype, we found a marginally significant increase in DBP at the pain threshold, b = 8.03, SE = 4.42, 95%CI [−0.64, 16.70], t = 1.82, *p* = 0.069, while the increase at pain tolerance was significant, b = 9.68, SE = 4.42, 95%CI [1.01, 18.35], t = 2.19, *p* = 0.029.

## 3. Discussion

The CPT is one of the most commonly utilized protocols for inducing stress in human research [9,10,11,12]. It consistently triggers a stress response characterized by robust activation of the sympathetic nervous system (SNS) as well as the hypothalamic–pituitary–adrenal (HPA) [11,13,14]. Activation of the SNS during exposure to cold leads to the release of many potent biologically active neurotransmitters and hormones, such as catecholamines (primary norepinephrine, NE), as well as endothelins, prostaglandins, and angiotensin II [15]. This results in cardiovascular effects, including an increase in BP and HR, which are the most frequently reported indicators in human stress research [11,12,16,17]. It is estimated that genetic factors account for up to 50% of the variance in BP (both SBP and DBP), with the remaining variance attributed to psychological, psychosocial, and lifestyle factors [5,18,19]. Genome-wide association studies of BP have identified more than 1000 loci, and *ADRB2* gene polymorphic sites are among them [5]. In our study, we tested the hypothesis that rs1042713 (G>A, Gly16Arg) and rs1042714 (G>C, Glu27Gln) polymorphisms of the *ADRB2* gene influence SBP and DBP during CPT in young healthy men, including combat athletes. The general observation of our study was that athletes, on average, independent of their genotype, had higher SBP and DBP than students and that SBP and DBP were significantly higher, both in students and combat athletes, when measured at the time of pain tolerance than when measured before CPT. The significantly higher SBP and DBP observed in combat athletes compared to non-athletes may be attributed to the heightened sensitivity of the autonomic nervous system, which probably functions more effectively in these athletes than in non-athletes. The evaluation of psychological resilience, particularly the levels of stress and anxiety experienced by the participants during the CPT, should also be considered [17]. Athletes involved in contact sports, as opposed to those in non-contact sports, tend to exhibit higher levels of sensory excitability and are more prone to aggressive behavior [20,21]. Elevated anxiety levels, aggressive tendencies, and heightened reactivity can significantly impact the fundamental parameters of the cardiovascular system. No significant interaction was detected between the study group and measurement time, suggesting that the observed differences in the timing of SBP and DBP measurements were consistent across both groups. This finding indicates that the pain stimulus induced by the CPT results in similar functional changes in the cardiovascular systems of both athletes and non-athletes, as was reported in our previous study [17].

When we analyzed SBP and DBP in relation to the rs1042713 Gly16Arg polymorphic site in both groups, it turned out that combat athletes and non-athletes had different levels of SBP, but not DBP, during CPT depending on their genotype. The SBP of athletes did not vary according to their genotype; however, students’ SBP was significantly higher in Gly16Gly16 homozygotes when compared to 16Arg allele carriers. Moreover, students did not statistically differ from athletes in SBP if they had Gly16Gly16 genotypes but had lower SBP than athletes if they carried the 16Arg allele. The results of previous studies that investigated the effect of the *ADRB2* rs1042713 polymorphism on BP indices are not entirely conclusive. Several studies reported an association between BP, BP change, mean arterial pressure (MAP), or blood pressure variability (BPV) and the rs1042713 polymorphism [1,22,23,24]. A longitudinal study that aimed to elucidate the relevance of *ADRB2* polymorphisms to weight gain, BP elevation, and sympathetic nerve activity in young, non-obese, normotensive men indicated that carriers of the Gly16 allele were more susceptible to weight gain and BP elevation over the 5-year period [22]. In the next study, the authors tried to determine whether the Gly16Arg polymorphism differentially influenced cardiovascular function during short-duration, low- and high-intensity exercise [23]. The results suggested that subjects homozygous for 16Arg exhibited reduced MAP at rest, which persisted during exercise, with no evidence of differential changes throughout the exercise despite significant changes in catecholamine levels. The authors proposed that genotype-related differences in baseline receptor function or density might cause phenotypic differences at rest, which are maintained during short-term exercise [23]. Another study assessed the associations between the *ADRB2* Gly16Arg polymorphism and cardiovascular reactivity to both cold stress (forehead cold pressor test, CPT, and whole-body cold exposure, CE) and psychological stress (mental arithmetic, MA, and video game, VG) in healthy, normotensive Black adolescents and young adults [24]. They found that 16Arg16Arg homozygotes exhibited diminished DBP reactivity during the 3 min VG test compared to carriers of the Gly16 allele [24]. The most recent available study aimed to identify potential associations between the Gly16Arg polymorphism and various cardiovascular measures at rest, during orthostatic stress, and under cognitive load in a cohort of healthy young subjects [1]. They found significant associations with BPV indices—carriers of allele Gly16 had increased BPV magnitude in the low-frequency (LF) band (LF SBP and LF DBP) and overall BPV during rest and head-up tilt (HUT) phases of examination protocol [1]. Some studies have not demonstrated any significant association between BP, MAP, or BPV and the rs1042713 polymorphism [25,26]. Additionally, contrasting results were observed in a study by Castellano et al. [2] and Busjahn et al. [27] in which the 16Arg allele was associated with higher SBP in normal, healthy mono- and di-zygotic twin subjects as well as in individuals from the general population under the age of 50.

When SBP and DBP were analyzed in relation to the rs1042714 Glu27Gln polymorphic site in both groups, we found that combat athletes and non-athletes exhibited different levels of SBP, but not DBP, during the CPT depending on their genotype. The SBP of students did not vary according to genotype; however, athletes carrying the Glu27 allele had significantly higher SBP compared to 27Gln27Gln homozygotes. Furthermore, athletes with the 27Gln27Gln genotype did not show a significant difference in SBP compared to students, whereas athletes carrying the Glu27 allele had higher SBP than students with the same allele. We also identified an interaction between the timing of SBP measurement and the rs1042714 polymorphism. The analysis revealed that both students and combat athletes with the 27Gln27Gln genotype exhibited similar SBP levels regardless of the timing during the CPT. In participants with the Glu2727Gln genotype, significant increases in SBP were observed both at the pain threshold and pain tolerance points, while in those with the Glu27Glu27 genotype, a significant increase was noted only at the pain tolerance point. Similarly to the Gly16Arg polymorphism, the association between the Glu27Gln polymorphism and BP remains unclear. Several studies have investigated the impact of the *ADRB2* rs1042714 polymorphism on BP indices, with some identifying a link between the Glu27 allele and the Glu27Glu27 genotype and elevated BP, MAP, and BPV. The results of one study showed that individuals carrying the Glu27 allele were more commonly among those who experienced significant weight gain or BP elevation over the 5-year study period [22]. Moreover, among those who experienced significant weight gain, individuals with concurrent significant BP elevation had a higher frequency of both the Gly16 and Glu27 alleles [22]. Another also investigated the associations between cardiovascular reactivity to CPT and CE as well as MA, VG, and Glu27Gln polymorphism [24]. The marginal interaction of the Glu27Gln and DBP was found—greater DBP reactivity during VG in Glu27Glu27 homozygotes than in carriers of the Gln27 allele has been noted [24]. In contrast to the previously mentioned findings, a study by Castellano et al. [2] demonstrated that elevated BP values were more specifically associated with the presence of the Arg16-Gln27 haplotype. Additionally, younger individuals carrying the Arg16-Gln27 haplotype exhibited a trend toward lower HR, higher BMI, lower glycemia, and elevated triglyceride levels [2].

In vitro studies investigating the functional consequences of the N-terminal rs1042713 and rs1042714 polymorphisms suggest that the Gly16Arg and Glu27Gln variants influence β2-AR function, though findings in this area have been inconsistent [26,27,28]. Green et al. [28] utilized Chinese hamster fibroblasts (CHW) to assess the functional implications of the Gly16Arg and Glu27Gln variants. Their research indicated that the Gly16 variant was associated with enhanced agonist-induced desensitization, whereas the Glu27 allele was linked to resistance to desensitization compared to the Arg16 and Gln27 alleles [28]. These results were further supported in primary cultures of human airway smooth muscle cells (HASMC), where cells expressing the Gly16 β2-AR exhibited enhanced agonist-promoted downregulation, while those expressing the Glu27 β2-AR were relatively resistant to such downregulation following prolonged (24 h) exposure to the β2-AR agonist isoproterenol [29]. The opposite results were obtained by Koryakina et al. [30], who employed site-directed mutagenesis and recombinant expression in human embryonic kidney cells (HEK-293) to investigate the functional consequences of the four possible combinations of Gly16Arg and Glu27Gln polymorphisms when exposed to isoprenaline treatment. The study found that isoforms containing the Arg16 variant (both 16Arg27Gln and 16ArgGlu27) exhibited increased down-regulation, as indicated by a reduction in receptor binding sites. Furthermore, isoforms with the Gly16 β2-AR variant (Gly16Gln27 and Gly16Glu27) were relatively resistant to down-regulation. The Arg16Gln27 β2-AR demonstrated the most pronounced reduction in receptor number, suggesting that the glutamic acid residue at position 27 has a greater propensity for agonist-induced down-regulation when paired with the arginine residue at position 16. However, any potential effect of the Glu27Gln to enhance down-regulation is mitigated by the dominant influence of the glycine residue at position 16. It was assumed that the described discrepancies between studies were hypothesized to be attributed to differences in the expression systems [28,29,30].

Studies examining the effects of *ADRB2* polymorphisms on vascular reactivity and desensitization in healthy subjects following β2-AR agonist stimulation have also produced inconclusive results. Scientists investigated agonist-mediated in vivo vasodilation in normotensive Austrian Caucasians [31]. The study demonstrated that individuals with the Gly16Gly16 β2-AR genotype had significantly higher resting MAP compared to those with the 16Arg16Arg genotype. Moreover, homozygous Gly16 subjects exhibited significantly reduced vasodilation during salbutamol infusion compared to 16Arg16Arg individuals. These findings suggest that the Arg16 allele is associated with a greater vasodilatory response, whereas the Gly16 allele may lead to increased agonist-induced downregulation [31]. A study by Dishy et al. [32] aimed to assess the effects of the *ADRB2* Gly16Arg and Glu27Gln polymorphisms on agonist-induced venodilation and desensitization in the human vasculature. The findings revealed that individuals homozygous for 16Arg and 27Gln exhibited enhanced agonist-mediated desensitization, as measured by vascular responses in the dorsal hand vein, whereas those homozygous for Gly16 (regardless of the amino acid at position 27) showed resistance to agonist-mediated desensitization. Additionally, the study found that individuals homozygous for Glu27 had an enhanced vasodilatory response to isoproterenol.

β2-ARs mediate physiological responses such as increased perfusion and vasodilation. The vasodilatory effect of epinephrine (EPI), a β2-AR agonist, typically counteracts the BP increase induced by its α-adrenergic stimulation [32]. The Gly16Arg and Glu27Gln polymorphisms are associated with variable responses to agonist stimulation, resulting in differing degrees of desensitization and downregulation [31,33]. Both in vitro and in vivo studies suggest that the presence of β2-AR isoforms encoded by alleles that enhance receptor desensitization and subsequent downregulation in response to agonist action also concurrently reduce vasodilatory effects [28,30,31]. The modulation of vascular desensitization and downregulation has significant clinical implications for responses to SNS stimulation, both under physiological conditions (e.g., exercise, stress) and in pathological states such as hypertension [32].

## 4. Materials and Methods

### 4.1. Ethics Statement

The study was approved by the Bioethics Committee of the Regional Medical Chamber in Szczecin (No. 09/KB/V/2013).

The study protocols were conducted ethically in accordance with the Declaration of Helsinki of the World Medical Association and the Declaration on Strengthening the Reporting of Genetic Association Studies (STREGA). Participants were informed of the risks and benefits of the experimental protocols, and each participant completed a written informed consent form. All participants gave their written consent to participate in the study. All personal information and results were anonymized.

### 4.2. Participants

We recruited N = 379 healthy men aged 18 to 43 years. The experimental group consisted of 203 martial artists (age M = 24.73, SD = 6.65; 2 of them did not provide demographic information) who had at least five years of experience in boxing (n = 95), karate (n = 82), and other martial arts (n = 26). The comparison group consisted of 176 unrelated university students (age M = 21.13, SD = 1.88, 1 of whom did not provide demographic information) who did not participate in sports at a professional level.

### 4.3. Procedure and Measurements

The cold pressor test (CPT) is a standard laboratory technique for measuring pain threshold and pain tolerance. Before participating in the study, all participants were informed about the study procedure.

At the beginning of the CPT, the participant immersed his right hand up to the wrist in a box of 37 °C warm water. The hand remained in the warm water for 2 min to normalize the skin temperature [34].

The participant then placed the hand in a glass box containing ice water at a temperature between 0 °C and 0.5 °C. The test subject indicated “pain” when they felt pain in their hand for the first time (pain threshold) and when the pain became unbearable (pain tolerance).

Pain threshold and pain tolerance were measured in seconds. The maximum time the hand remained in the box was 120 s, but participants were not informed of this endpoint.

Cardiovascular measurements taken during the study, including blood pressure and heart rate, were recorded at three time points: (1) before the study, (2) while the hand was immersed in cold water when the subject felt pain, and (3) at the end of the study when the subject removed their hand from the box—maximum pain.

Heart rate and blood pressure were recorded with a cuff on the left arm (the right arm was immersed in water). Measurements were taken continuously every 30 s using an Omron type 2 device (Omron M2Basic, Wroclaw, Poland). All measurements were taken in a sitting position.

### 4.4. Genotyping

Genomic DNA was extracted from the buccal cells by a Genomic Micro AX SWAB Gravity (A&A Biotechnology, Gdansk, Poland) according to the producer’s protocol. All samples were genotyped in duplicate using the TaqMan^®^ pre-designed SNP genotyping assays C___2084764_20 for the rs1042713 and C___2084765_20 for the rs1042714 (Applied Biosystems, Foster City, CA, USA) according to the manufacturer’s protocol in the CFX96 Touch Real-Time PCR Detection System (Biorad, Hercules, CA, USA).

**Figure 5:**
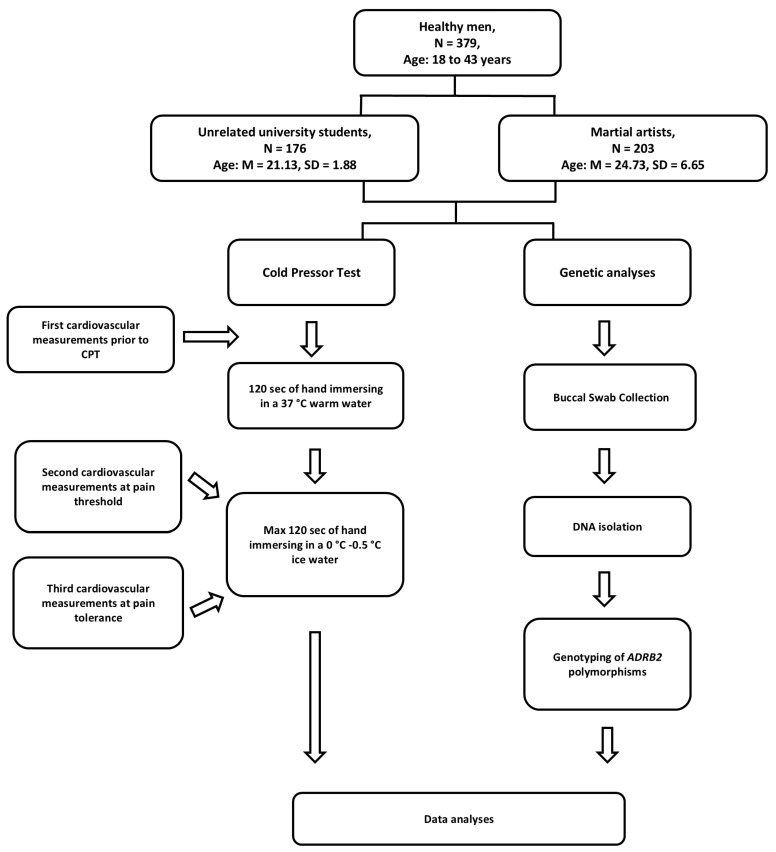
Flowchart of the experimental protocol.

The research protocol diagram is presented in Figure 5.

### 4.5. Statistical Analyses

Data analysis was performed with JAMOVI 2.6.23 (descriptive statistics and mixed linear regression) [35].

The threshold for statistical significance was set at *p* < 0.05. The distributions of SBP and DBP deviated significantly from a normal distribution (*p* < 0.001), so they were right-skewed. Therefore, we used the U Mann–Whitney test for a two-group comparison. For the remaining analyses, the data were considered nested because all participants underwent blood pressure measurements three times at different time points of the CPT task. Therefore, we used multilevel modeling with JAMOVI with REML estimation, which allows the use of variables that deviate from the normal distribution.

The mixed model regressions included the following independent variables: (1) group (effect coded with students as the reference group); (2) measurement point during the CPT (pre-test, pain threshold, pain tolerance), effect coded with pre-test measurement as reference; (3) genotype of *ADRB2* rs1042713 polymorphism (GG, AG, AA), effect coded with GG genotype as reference group; and (4) genotype of *ADRB2* rs1042714 polymorphism (CC, GC, GG), effect coded with CC genotype as reference group. The regression also included interactions between the above variables and included a random intercept for participants. Regression analysis was performed twice for systolic and diastolic blood pressure as dependent variables.

## 5. Conclusions

In conclusion, in the Polish population, the Gly16 and Glu27 alleles appear to be associated with an increase in BP in response to stress. The elevated BP levels observed in our study among combat athletes compared to students are likely attributable to heightened autonomic nervous system sensitivity. In these athletes, heightened sensitivity appears to be influenced by the Glu27 allele of the Glu27Gln polymorphism rather than by the Gly16Arg polymorphism. Our analysis of the relationship between BP and *ADRB2* gene polymorphisms in non-athletes suggests an association between SBP and the Gly16Arg polymorphism, with Gly16Gly16 homozygosity seemingly predisposing young, healthy, non-trained men to elevated BP.

## 6. Limitations

This study has several limitations that need to be acknowledged. One limitation is the absence of measurements for potential confounding factors such as diet, lifestyle, environmental stress, and hormonal profiles, all of which could influence SBP, DBP, and HR responses during the CPT. Additionally, the study was conducted exclusively on young, healthy males from two specific groups—combat sports athletes and non-athlete university students—limiting the generalizability of the results to other populations, including females and individuals with different health statuses. Furthermore, only two *ADRB2* gene polymorphisms (rs1042713 and rs1042714) were analyzed, while other potential genetic causal variants and candidate effector genes for BP as well as gene-environment interactions that may affect cardiovascular responses were not considered. Lastly, the study’s sample size, although sufficient for preliminary analysis, may limit the power to detect more subtle genetic associations.

## Figures and Tables

**Table 1:** Demographic and anthropometric data and the results from the CPT in the combat athletes and control group.

Variables	Combat Athletes		Comparison Group		U Statistics	*p*	Effect Size
	M	SD	M	SD			Cohens’s d
Age (years)	24.73	6.65	21.13	1.88	13,884.0	**0.001**	0.21
Height (m)	1.79	0.07	1.82	0.08	13,713.5	**0.001**	0.22
Body Mass (kg)	78.30	12.96	77.86	9.66	17,342.5	0.816	0.01
BMI (kg/m^2^)	24.47	3.28	23.55	2.16	14,808.0	**0.008**	0.16
CPT 1	25.12	27.41	26.62	23.96	16,612.5	0.231	0.07
CPT 2	98.89	31.63	86.22	34.81	13,995.0	**0.001**	0.22

Note: Effect size—rank biserial correlation, significance level *p* ≤ 0.05 in bold.

**Table 2:** Results of a multilevel regression examining SBP as a function of study group, pain threshold, and pain tolerance using the CPT and the different genotypes of *ADRB2* polymorphisms rs1042713 and rs1042714.

Names	Effect	b	SE	Lower	Upper	df	t	*p*
**(Intercept)**	**(Intercept)**	**139.70**	**0.87**	**137.99**	**141.42**	**369**	**159.94**	**0.001**
**Group**	**athletes–students**	**8.83**	**1.75**	**5.40**	**12.26**	**369**	**5.05**	**0.001**
Measurement	at pain threshold–pre-test	1.02	0.71	−0.38	2.42	738	1.43	0.153
	**at pain tolerance–pre-test**	**2.63**	**0.71**	**1.22**	**4.03**	**738**	**3.68**	**0.001**
rs1042713	AG–GG	−0.29	1.75	−3.72	3.15	369	−0.16	0.870
	AA–GG	−2.59	2.71	−7.91	2.73	369	−0.96	0.340
rs1042714	CG–CC	1.70	1.90	−2.03	5.43	369	0.89	0.372
	GG–CC	2.55	2.65	−2.66	7.75	369	0.96	0.338
Group × Measurement	(athletes–students) × (at pain threshold–pre-test)	1.71	1.43	−1.09	4.51	738	1.20	0.232
	(athletes–students) × (at pain tolerance–pre-test)	2.00	1.43	−0.81	4.80	738	1.40	0.163
**Group** **× rs1042713**	**(athletes–students)** **×** **(AG–GG)**	**7.05**	**3.50**	**0.17**	**13.92**	**369**	**2.01**	**0.045**
	**(athletes–students)** **×** **(AA–GG)**	**11.91**	**5.42**	**1.27**	**22.54**	**369**	**2.20**	**0.029**
Measurement × rs1042713	(at pain threshold–pre-test) × (AG–GG)	0.19	1.43	−2.62	3.00	738	0.14	0.892
	(at pain tolerance–pre-test) × (AG–GG)	1.14	1.43	−1.67	3.95	738	0.80	0.427
	(at pain threshold–pre-test) × (AA–GG)	0.20	2.22	−4.15	4.55	738	0.09	0.927
	(at pain tolerance–pre-test) × (AA–GG)	0.92	2.22	−3.43	5.26	738	0.41	0.680
Group × rs1042714	(athletes–students) × (CG–CC)	7.15	3.80	−0.30	14.61	369	1.88	0.061
	**(athletes–students)** × **(GG–CC)**	**11.67**	**5.31**	**1.26**	**22.08**	**369**	**2.20**	**0.029**
**Measurement** × **rs1042714**	(at pain threshold–pre-test) × (CG–CC)	2.79	1.55	−0.26	5.84	738	1.80	0.073
	**(at pain tolerance–pre-test)** × **(CG–CC)**	**3.47**	**1.55**	**0.42**	**6.51**	**738**	**2.23**	**0.026**
	(at pain threshold–pre-test) × (GG–CC)	2.64	2.17	−1.62	6.90	738	1.22	0.225
	**(at pain tolerance–pre-test)** × **(GG–CC)**	**4.87**	**2.17**	**0.61**	**9.13**	**738**	**2.25**	**0.025**
Group × Measurement × rs1042713	(athletes–students) × (at pain threshold–pre-test) × (AG–GG)	1.84	2.86	−3.78	7.46	738	0.64	0.520
	(athletes–students) × (at pain tolerance–pre-test) × (AG–GG)	−0.12	2.86	−5.74	5.50	738	−0.04	0.968
	(athletes–students) × (at pain threshold–pre-test) × (AA–GG)	2.90	4.43	−5.80	11.59	738	0.65	0.514
	(athletes–students) × (at pain tolerance–pre-test) × (AA–GG)	0.41	4.43	−8.29	9.10	738	0.09	0.927
Group × Measurement × rs1042714	(athletes–students) × (at pain threshold–pre-test) × (CG–CC)	2.12	3.11	−3.97	8.22	738	0.68	0.495
	(athletes–students) × (at pain tolerance–pre-test) × (CG–CC)	−2.36	3.11	−8.45	3.74	738	−0.76	0.448
	(athletes–students) × (at pain threshold–pre-test) × (GG–CC)	1.90	4.34	−6.62	10.41	738	0.44	0.662
	(athletes–students) × (at pain tolerance–pre-test) × (GG–CC)	−4.40	4.34	−12.91	4.12	738	−1.01	0.311

Note: significance level *p* ≤ 0.05 in bold.

**Table 3:** Results of a multilevel regression examining DBP as a function of study group, pain threshold, and pain tolerance using the CPT and the different genotypes of *ADRB2* polymorphisms rs1042713 and rs1042714.

Names	Effect	b	SE	Lower	Upper	df	t	*p*
(Intercept)	(Intercept)	77.52	0.98	75.60	79.44	369	79.30	0.001
**Group**	**athletes–students**	**4.17**	**1.96**	**0.33**	**8.00**	**369**	**2.13**	**0.034**
**Measurement**	**at pain threshold–pre-test**	**5.06**	**1.86**	**1.41**	**8.72**	**738**	**2.72**	**0.007**
	**at pain tolerance–pre-test**	**5.32**	**1.86**	**1.67**	**8.98**	**738**	**2.86**	**0.004**
rs1042713	AG–GG	0.61	1.96	−3.24	4.45	369	0.31	0.758
	AA–GG	−3.81	3.03	−9.76	2.14	369	−1.26	0.210
rs1042714	CG–CC	−2.50	2.13	−6.68	1.67	369	−1.18	0.240
	GG–CC	−0.60	2.97	−6.43	5.23	369	−0.20	0.839
Group × Measurement	(athletes–students) × (at pain threshold–pre-test)	−1.71	3.73	−9.02	5.60	738	−0.46	0.646
	(athletes–students) × (at pain tolerance–pre-test)	−0.30	3.73	−7.61	7.01	738	−0.08	0.935
Group × rs1042713	(athletes–students) × (AG–GG)	4.58	3.92	−3.11	12.27	369	1.17	0.244
	(athletes–students) × (AA–GG)	1.97	6.07	−9.93	13.88	369	0.33	0.745
Measurement × rs1042713	(at pain threshold–pre-test) × (AG–GG)	−1.69	3.73	−9.02	5.63	738	−0.45	0.650
	(at pain tolerance–pre-test) × (AG–GG)	−0.71	3.73	−8.04	6.62	738	−0.19	0.850
	(at pain threshold–pre-test) × (AA–GG)	8.46	5.78	−2.88	19.80	738	1.46	0.144
	(at pain tolerance–pre-test) × (AA–GG)	10.42	5.78	−0.92	21.75	738	1.80	0.072
Group × rs1042714	(athletes–students) × (CG–CC)	0.56	4.25	−7.78	8.91	369	0.13	0.895
	(athletes–students) × (GG–CC)	1.54	5.94	−10.12	13.20	369	0.26	0.796
Measurement × rs1042714	**(at pain threshold–pre-test)** × **(CG–CC)**	**10.68**	**4.05**	**2.73**	**18.63**	**738**	**2.64**	**0.009**
	**(at pain tolerance–pre-test)** × **(CG–CC)**	**11.63**	**4.05**	**3.68**	**19.58**	**738**	**2.87**	**0.004**
	(at pain threshold–pre-test) × (GG–CC)	9.79	5.66	−1.32	20.89	738	1.73	0.084
	**(at pain tolerance–pre-test)** × **(GG–CC)**	**12.35**	**5.66**	**1.25**	**23.46**	**738**	**2.18**	**0.029**
Group × Measurement × rs1042713	(athletes–students) × (at pain threshold–pre-test) × (AG–GG)	−3.35	7.47	−18.01	11.30	738	−0.45	0.654
	(athletes–students) × (at pain tolerance–pre-test) × (AG–GG)	0.77	7.47	−13.88	15.43	738	0.10	0.917
	(athletes–students) × (at pain threshold–pre-test) × (AA–GG)	9.03	11.56	−13.65	31.71	738	0.78	0.435
	(athletes–students) × (at pain tolerance–pre-test) × (AA–GG)	9.24	11.56	−13.44	31.92	738	0.80	0.424
Group × Measurement × rs1042714	(athletes–students) × (at pain threshold–pre-test) × (CG–CC)	7.08	8.10	−8.82	22.97	738	0.87	0.383
	(athletes–students) × (at pain tolerance–pre-test) × (CG–CC)	5.43	8.10	−10.46	21.33	738	0.67	0.503
	(athletes–students) × (at pain threshold–pre-test) × (GG–CC)	5.35	11.32	−16.85	27.56	738	0.47	0.636
	(athletes–students) × (at pain tolerance–pre-test) × (GG–CC)	5.20	11.32	−17.00	27.41	738	0.46	0.646

Note: significance level *p* ≤ 0.05 in bold.

## Data Availability

The data that support the findings of this study are available from the corresponding author upon reasonable request.
